# LONG-TERM EXPOSURE TO OUTDOOR LIGHT AT NIGHT, AIR POLLUTION, AND DEPRESSIVE SYMPTOMS AMONG OLDER PEOPLE IN ENGLAND

**DOI:** 10.1093/geroni/igae098.1250

**Published:** 2024-12-31

**Authors:** Rina So, Giorgio Di Gessa, Shaun Scholes, Su-Hwan Kim, Yujin Park, Jinkook Lee, Sara Adar, Paola Zaninotto

**Affiliations:** University College London, London, England, United Kingdom; University College London, London, England, United Kingdom; University College London, London, England, United Kingdom; Gyeongsang National University, Jinju-Si, Kyongsang-namdo, Republic of Korea; Seoul National University Hospital, Seoul, Republic of Korea; University of Southern California, Los Angeles, California, United States; University of Michigan, Ann Arbor, Michigan, United States; University College London, London, England, United Kingdom

## Abstract

Air pollution and light at night (LAN) are environmental stressors that may lead to depression, but the epidemiological evidence is inconsistent and scarce, especially for LAN. We used data from 8,583 participants (25,151 observations), aged ≥ 50 years, of the English Longitudinal Study of Ageing from waves 6-9 (2012-2019). Annual levels of fine particulate matter (PM2.5), nitrogen dioxide (NO2), and LAN were assessed at participants’ residential addresses for each interview year. Having depressive symptoms was defined as reporting ≥3 items of the 8-item Center for Epidemiologic Studies Depression Scale. We used mixed effects generalized linear models (binomial distribution) to examine associations of air pollutants and LAN (in quartiles) with depressive symptoms, adjusting first for age and sex and then adding control for individual lifestyle and socio-demographic factors, and area-level deprivation index. In models minimally-adjusted, increased odds of having depressive symptoms were observed for PM2.5, NO2, and LAN with odds ratio (OR) (95% confidence interval) of 1.15 (1.05, 1.25) and 1.20 (1.10, 1.32) per interquartile range of PM2.5 (2.2 µg/m3) and NO2 (4.2 ppb), respectively, and 2.47 (1.96, 3.12) in the highest quantile of LAN compared to the lowest. These associations were attenuated in fully-adjusted models to 0.97 (0.90, 1.05) for NO2 and 0.91 (0.84, 0.99) for LAN, while the association with PM2.5 remained positive [OR: 1.05 (0.97, 1.14)]. Higher residential PM2.5 levels might be associated with an increased risk of having depressive symptoms, but associations with NO2 and LAN were explained by confounding by individual- and area-level characteristics.

